# The association between preoperative anxiety and chronic post-surgical pain after general anaesthesia, a systematic review and meta-analysis

**DOI:** 10.1016/j.bjao.2025.100487

**Published:** 2025-10-06

**Authors:** Mirjam Bakker-Bons, Ria M.J. Hijmering, Remko Soer, André P. Wolff

**Affiliations:** 1 University of Groningen, University Medical Center Groningen, Department of Anesthesiology, Groningen, the Netherlands; 2 University of Groningen, University Medical Center Groningen, Department of Anesthesiology, UMCG Pain Center, Haren, the Netherlands; 3 mProve hospitals. A strategic cooperation between seven Dutch top-clinical hospitals, Zwolle, the Netherlands

**Keywords:** chronic pain, general anaesthesia, meta-analysis, preoperative anxiety, post-surgical, systematic review

## Abstract

**Background:**

Chronic post-surgical pain (CPSP) is a burden for both patients and healthcare, yet current treatment options are insufficient. Previous studies indicate preoperative anxiety as a risk factor for developing CPSP, yet no high-quality review exists. This study aims to systematically review the relationship between increased preoperative anxiety and the incidence of new CPSP.

**Methods:**

Four databases were used to identify relevant studies for a systematic review and meta-analysis. Inclusion criteria included adult patients undergoing surgical procedures under general anaesthesia, measuring preoperative anxiety with validated tools, and postoperative pain at least 3 months after surgery. Preferred Reporting Items for Systematic reviews and Meta-Analyses (PRISMA) guidelines were followed, and a risk of bias analysis was performed.

**Results:**

Of the 233 studies retrieved, 26 studies were included in the systematic review. Following risk of bias analysis, 23 papers were included in the meta-analysis. A correlation was found between preoperative anxiety and CPSP, with a standardised mean difference of 0.31 (95% confidence interval 0.20–0.41). High heterogeneity was observed, which was attributed to several possible confounding factors. Subgroup analysis did not alter this outcome. When translating the outcome to a relevant scale, we observed an increase in numeric rating scale pain of 0.34 for patients experiencing preoperative anxiety.

**Conclusions:**

There is moderate-quality evidence indicating a positive association between preoperative anxiety and CPSP, where an increase in preoperative anxiety correlates with an increased incidence of CPSP. More research is needed to identify specific patients that would benefit from treating preoperative anxiety and thus potentially preventing CPSP.

**Systematic review protocol:**

PROSPERO (CRD42024513479).

**Editor’s key points**

• Chronic post-surgical pain (CPSP), defined as new or increased pain after surgery that persists for a least 3 months postoperatively, is a universal unaddressed problem in healthcare.
• This report identifies a correlation between preoperative anxiety and increased risk of CPSP in adult patients after elective surgery, however the high amount of heterogeneity limits interpretation of clinical relevance.

Chronic pain is a major public health issue^1^ associated with a reduced quality of life and high economic costs because of (work) disability and increased use of healthcare.^2^, ^3^, ^4^, ^5^ One of the seven categories of chronic pain, as described by the International Code of Diseases (ICD-11^6^), is chronic post-surgical pain (CPSP). CPSP is defined as new or increased pain after surgery that persists for at least 3 months post surgery, without the presence of other causes of pain such as infection, tumour recurrence, or pre-existing conditions.^3^, ^4^, ^5^, ^6^, ^7^^,^^7^ The incidence of CPSP varies by type of surgery, with an average range of 10–75%.^5^^,^^7^ In the majority of cases, treatment and prevention through perioperative pharmacological intervention remain a challenge in CPSP.^5^ A promising approach is looking at preoperative factors influencing chronic pain. The neuroanatomical basis of psychological stress could play a role in modulating pain.^8^ Furthermore, the association between psychological stress and a chronic inflammatory response could play a role in the impairment of normal post-surgical healing.^9^ Therefore, the relationship between preoperative anxiety and CPSP is of particular interest.^10^

The prevalence of any form of preoperative anxiety in patients is between 85% and 93%,^11^ with anxiety most frequently cited as the worst aspect of the perioperative experience.^12^^,^^13^ Higher levels of anxiety correlate with poorer outcomes, including increased pain intensity and postoperative chronic pain.^14^, ^15^, ^16^, ^17^, ^18^ Gaining a better understanding of the relationship between anxiety and CPSP may lead to better preventive actions, such as the application of education or stress-reducing therapies, including cognitive behavioural interventions before surgery for those who are at high risk of developing CPSP. A systematic review and meta-analysis^15^ previously examined the relationship between several preoperative psychological factors and the risk of developing CPSP. However, since 2012, substantial additional research has been conducted, highlighting the need for an updated review that specifically focuses on preoperative anxiety.

This meta-analysis will follow the current methodology as described by the Cochrane Group.^19^ One challenge is the presence of numerous tools for diagnosing anxiety, which impacts the reliability and comparability of results. We aim to enhance confidence in the findings by applying strict inclusion criteria for anxiety and chronic pain. Furthermore, to improve the clinical relevance of this meta-analysis, the outcomes will be translated back into a clinically relevant scale.^20^ The purpose of this systematic review and meta-analysis is to study the association between the presence of preoperative anxiety and the incidence of new CPSP and to assess the clinical relevance of this association.

## Methods

A systematic review and meta-analysis were performed according to the Preferred Reporting Items for Systematic reviews and Meta-Analyses (PRISMA) 2020 statement.^21^ The updated 27-item checklist was used as a guideline. The protocol was prospectively registered with PROSPERO on 16 February 2024 (CRD42024513479).

### Literature search

The following databases were included in the literature search: PubMed, EMBASE, PsychINFO, and the Cochrane Library. The search was limited to articles published up to October 2024. Title, abstract, keywords, or full text were searched using the following search terms: ‘anxiety’, ‘preoperative anxiety’, ‘mental distress’, ‘pain’, ‘postoperative pain’, ‘chronic pain’, ‘surgical’, ‘anaesthesia’, ‘adult’ (see Supplementary Table S1 for complete search strategy).

Records identified from the databases were retrieved manually, and duplicates were removed using Rayyan.ai.^22^ Records were independently screened by authors MBB, RH, and RS for usability based on title and abstract. Commentaries, study protocols, case reports, and animal studies were excluded. Review papers were excluded after consultation of the reference lists for relevant citations. After excluding irrelevant records, reports were retrieved for a full-text review to confirm eligibility. Disagreements were resolved through discussion between the authors; in cases where no agreement was reached, a decision was made by a third reviewer (AW). Interrater reliability was assessed using Cohen’s kappa.^23^

### Inclusion and exclusion criteria

Studies were included if they met the following inclusion criteria, based on a predefined PECO (Patient, Exposure, Comparison, and Outcome) framework. The *population* consisted of adult patients (18 yr and older) who underwent surgery, received general anaesthesia, and were not diagnosed with an anxiety disorder. The *exposure* included the presence of preoperative anxiety, which was *compared* with patients with no preoperative anxiety. To exclude a possible effect of intraoperative anxiety, patients receiving local or regional anaesthesia were excluded. Preoperative anxiety can be measured using many different tools.^24^ To increase comparability between studies and reduce heterogeneity, only studies that used validated measurement tools were included.^25^ These tools utilised multiple questions to determine the presence of anxiety, ensuring a reliable and valid measurement. The *outcome* was the presence of chronic pain. Following the ICD-11 definition, chronic pain is any pain that persists after surgery for longer than 3 months.^6^ To be eligible for inclusion, the presence of chronic pain was assessed using prescribed tools that measure pain intensity or severity.^26^ Patients with pre-existing chronic pain were excluded to avoid any potential influence on CPSP.

### Data extraction

The following data were manually extracted from each paper: type of study, geographical location of the study, sample size, type of surgery, anxiety measurement tool, pain measurement tool, statistical methods, and statistical outcomes. When data of interest were presented only graphically, the data were extracted using PlotDigitizer version 3.1.5 (2023). The extracted data were manually checked before analysis to prevent errors.

### Risk of bias

To assess the certainty of the overall evidence, the risk of bias was calculated, using the Risk of Bias In Non-randomized Studies of Interventions (ROBINS-I), as described by Sterne and colleagues.^27^ For each paper seven different domains were judged on bias: bias related to confounding, selection of participants, classification of interventions, deviations from intended interventions, missing data, measurement of outcomes, and selection of the reported results. The overall risk was defined as serious when a study scored ‘serious risk’ in one or more domains. Results were visualised using the RoBVis program.^28^

### Statistics

Following Borenstein and colleagues,^29^ data were transformed to standardised mean differences (SMDs) to compare the data correctly and to use both continuous and correlational data,^30^ Cohen’s *d* was calculated with a corresponding standard error (se) for the SMD, using the algorithm from Wilson.^31^ When means and standard deviations were given, the SMD was calculated directly.^32^ For both odds ratio and correlation (*r*), the formulas described by Borenstein and colleagues^29^ and Chinn^33^ were used. A meta-analysis of the SMDs was performed using the Review Manager software from the Cochrane Collaboration (2022, version 5.4.1). A random-effects meta-analysis approach was used, as the estimated effect across different studies followed a random distribution. Forest plots were constructed with corresponding *I*^2^ values. Tau^2^ and *P*-values were calculated to assess heterogeneity. An outcome with an effect size ranging from 0.2 to 0.5 was considered a small effect size, from 0.5 to 0.8 a medium effect, and above 0.8 a large effect.^34^ Clinical relevance was calculated by translating the outcome back to the measurement scale, which involved multiplying the SMD by the standard deviation.^20^ Following the Cochrane Group, the standard deviation of the pain measurement scale used was taken from the largest and most representative study.^35^

## Results

### Literature search

**Fig 1:**
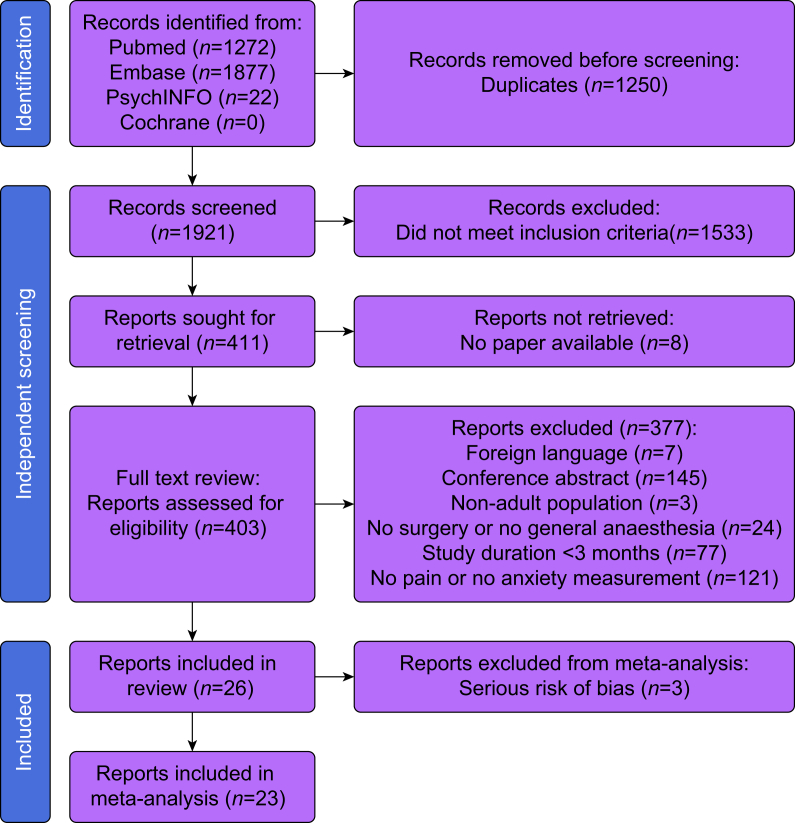
Workflow of literature search results and selection of eligible papers. Design based on PRISMA 2020 statement.^21^ PRISMA, Preferred Reporting Items for Systematic reviews and Meta-Analyses.

A visualisation of the selection process of eligible papers is given in Figure 1. The literature search yielded 1921 records, of which 26 studies were ultimately included in this review.^36^, ^37^, ^38^, ^39^, ^40^, ^41^, ^42^, ^43^, ^44^, ^45^, ^46^, ^47^, ^48^, ^49^, ^50^, ^51^, ^52^, ^53^, ^54^, ^55^, ^56^, ^57^, ^58^, ^59^, ^60^, ^61^ The overall agreement between reviewers resulted in a Cohen’s kappa of 0.90. For measurement of preoperative anxiety, only the Hospital Anxiety and Depression Scale (HADS) and State-Trait Anxiety Inventory (STAI) met our inclusion criteria.

### Study characteristics

An overview of the 26 included studies is given in Table 1. A total of 6498 patients were included, with a range of 31–1065 patients and a median of *n*=125 patients. The publication dates of the included papers ranged from 1997 to 2024. Studies were conducted in a wide range of countries (*n*=13), and the follow-up duration after surgery ranged from 3 to 60 months.

**Table 1:** Summary of studies used, including important results.

First author	Year	Geographic location	Study design	Type of surgery	Study length (months)	Sample size	Pain tool	Anxiety tool	Statistical method	Outcome	*P*-value	RoB
De Groot^36^	1997	Netherlands	Prospective cohort study	Lumbar laminectomy	3	126	VAS	STAI	Pearson correlation	*r*=0.38	0.01	
Gerbershagen^37^	2009	Germany	Prospective cohort study	Nephrectomy	6†	35	NRS	HADS	Mann–Whitney	No pain 7.25 (3.46) CPSP 10.33 (1.15)	0.38	
Lautenbacher^38^	2010	Germany	Prospective cohort study	Thorax malformation	6∗	84	NRS	STAI	Regression weight	d=0.73	0.73	
Hegarty^39^	2012	Ireland	Prospective cohort study	Lumbar discectomy	3	53	VAS	HADS	Spearman’s correlation	*r*=0.07	0.19	
VanDen Kerkhof^40^	2012	Canada	Prospective cohort study	Gynaecologic surgery	6	76	NRS	STAI	Poisson regression	RR=2.4	<0.05	
VanDen Kerkhof ^41^	2012	Canada	Prospective cohort study	Abdominal surgery	6	433	NRS	STAI	χ^2^test	RR=2.2	<0.05	
Masselin-Dubois^42^	2013	France	Prospective cohort study	Mastectomy/knee arthroplasty	3	182	NRS	STAI	Logistic regression	OR=1.02	0.01	
Choinière^43^	2014	Canada	Prospective cohort study	Cardiac surgery	24∗	975	NRS	HADS	Logistic regression	OR=1.09	<0.05	
Grosen^44^	2014	Denmark	Prospective cohort study	Thorax malformation	6	31	NRS	STAI	Spearman’s correlation	*r*=0.07	n.s.	
Miaskowski^45^	2014	USA	Prospective cohort study	Mastectomy	6∗	398	NRS	STAI	anova	*F*=4.83	0.01	
Utrillas-Compaired^46^	2014	Spain	Prospective cohort study	Knee arthroplasty	12	202	VAS	HADS	χ^2^ test	Not anxious 6.9 (1.2) Anxious 6.7 (1.9)	0.89	
Thomazeau^47^	2016	France	Prospective cohort study	Knee arthroplasty	6∗	104	NRS	HADS	χ^2^ test	No pain 8.1 (4.9) CPSP 11.1 (4.9)	0.01	
Bierke^48^	2017	Germany	Prospective cohort study	Knee arthroplasty	12†	100	NRS	STAI	Mann–Whitney	No CPSP med 10 IQR 10 CPSP med 20 IQR 17	<0.05	
Cho^49^	2017	USA	Prospective cohort study	Shoulder arthroplasty	12∗	46	VAS	HADS	Pearson correlation	*r*=−0.099	0.51	
Han^50^	2017	China	Prospective cohort study	Hysterectomy	3	870	NRS	HADS	Logistic regression	OR=2.07	0.01	
Nishimura^51^	2017	Japan	Prospective cohort study	Mastectomy	6∗	57	VAS	HADS	Logistic regression	OR=1.63	0.01	
Skeppholm^52^	2017	Sweden	Prospective cohort study	Cervical decompression	24∗	136	VAS	HADS	Mann–Whitney	Not anxious 19.5 (23.2)	0.01	
										Anxious 34.9 (28.3)		
Horn-Hofmann^53^	2018	Germany	Prospective cohort study	Thorax malformation	3	104	NRS	STAI	Logistic regression	OR=0.997	n.s.	
Habib^54^	2019	USA	Prospective cohort study	Mastectomy	12∗	124	VAS	STAI	Wilcoxon test	No pain med 45 IQR 7 CPSP med 47 IQR 8	0.34	
Larbig^55^	2019	Germany	Prospective cohort study	Limb amputation	12∗	52	NRS	STAI	χ^2^ test	No pain 27.6 (12) CPSP 42.4 (12.5)	0.01	
Borges^56^	2020	Brazil	Prospective cohort study	Caesarean section	3	462	NRS	HADS STAI	Logistic regression	RR=1.03 (HADS) RR=1.04 (STAI)	0.07 (HADS) 0.01 (STAI)	
Jiménez Ortiz^57^	2020	Spain	Prospective cohort study	Knee arthroplasty	12	260	VAS	HADS	anova	Not anxious 5.3 (4.4) Anxious 5.4 (4.3)	0.02	
Hardy^58^	2022	France	Prospective cohort study	Knee, shoulder, hip arthroplasty	12∗	266	VAS	STAI	χ^2^ test	No pain 39.3 (11.8) CPSP 34.1 (10.7)	0.01	
Danielsen^59^	2023	Denmark	Prospective cohort study	Lobectomy	6	121	NRS	HADS	Pearson correlation	RR=1.03	0.15	
Jin^60^	2023	China	Prospective cohort study	Various surgeries	6†	1065	NRS	HADS	Logistic regression	OR=2.40	0.01	
Olsen^61^	2024	Norway	Prospective cohort study	Knee arthroplasty	60	136	NRS	HADS	Logistic regression	OR=1.14	0.04	

Outcome is given in mean (standard deviation) when possible; *P*-value as given in article; *P*<0.05 when exact value was not available.

anova, analysis of variance; CPSP, chronic post-surgical pain; HADS, Hospital Anxiety and Depression Scale; IQR, interquartile range; med, median; NRS, numeric rating scale; n.s., not significant (*P*-value not given in article); OR, odds ratio; RoB, risk of bias (see Supplementary Figure S1 for full bias table); RR, Risk Ratio; STAI, State Trait Anxiety Inventory; VAS, visual analogue scale.

Low Risk.

Moderate Risk.

Serious Risk.

∗ more timepoints measured in study, but only latest is presented in paper;

† more timepoints presented in paper, but only latest measurement used.

In 10 articles, postoperative pain was measured at various time points; however, only the results for the last measurement were presented.^38^^,^^43^^,^^45^^,^^47^^,^^49^^,^^51^^,^^52^^,^^54^^,^^55^^,^^58^^,^^62^^,^^63^ In three articles, data were presented for two separate time points at which chronic pain was scored.^37^^,^^48^^,^^60^ For these studies, only the latest time point is included in this meta-analysis, as is the case with articles that only present results for the latest time point.

All studies were performed on adult patients undergoing elective surgery under general anaesthesia. Most studies selected only a specific procedure, such as lumbar disc surgery, knee arthroplasty, or coronary artery bypass grafting. Two different medical procedures were included in one study,^42^ namely total knee arthroplasty and breast surgery. Another study did not select for a specific procedure and included all elective cases.^60^

Some articles accounted for potential confounders that could affect the outcome. Seventeen studies reported on age, with four showing a statistically significant effect: for two studies^42^^,^^46^ older age was associated with CPSP, but the other two studies^45^^,^^54^ found that younger age was associated with CPSP. Twelve studies examined the effect of sex, with two studies reporting a statistically significant result with women having a higher incidence of CPSP.^43^^,^^61^ Seven studies examined the impact of education level, with one reporting a statistically significant result where higher education status was associated with increased risk for CPSP.^47^ Lastly, 13 studies investigated overweight, with one study identifying a statistically significant effect where a higher body mass index was associated with CPSP.^45^ See Supplementary Table S2 for an overview.

Preoperative anxiety was assessed in 13 papers using the HADS measurement tool, while the remaining 12 papers used the STAI measurement tool. In one paper, both HADS and STAI were used to measure anxiety.^56^ Postoperative pain was scored using the numeric rating scale (NRS) in 17 papers and the visual analogue scale in nine papers.

A total of 16 papers reported a statistically significant association between preoperative anxiety and CPSP with low to moderate correlations (Table 1). For one paper, there might be an effect; Borges and colleagues^56^ found a difference between anxiety measured with STAI (Risk Ratio (RR)=1.04; *P*=0.01), compared with anxiety measured with HADS (RR=1.03; *P*=0.07). All studies with a statistically significant interaction between preoperative anxiety and CPSP showed a similar effect: patients with preoperative anxiety reported chronic pain more often compared with patients without preoperative anxiety.

### Risk of bias

An overview of the overall risk estimation for each paper is presented in Table 1; an extended overview per domain is available in Supplementary Figure S1. The overall risk of bias was low for one paper and serious for three papers. All other papers had an overall moderate risk of bias. For the three articles with a serious risk of bias, the issue was primarily attributable to the reported data. In the article by Bierke & Petersen^48^ the results were only presented in figures, and no numerical values were provided. The extraction of data yielded inconclusive results. In the article by Jiménez Ortiz and colleagues^57^ only the results of the statistical analysis were presented; however, recalculation of this analysis yielded a different result. The article by Larbig and colleagues^55^ only partially reported the results needed for the meta-analysis.

### Meta-analysis

**Fig 2:**
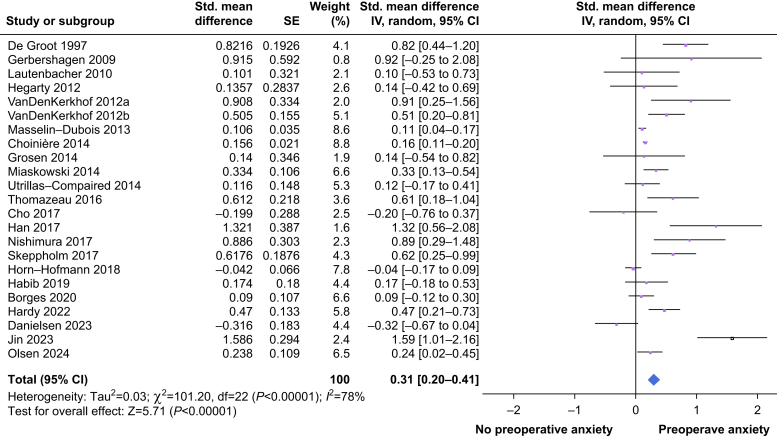
Forest plot of the meta-analysis. All standard mean differences (SMDs) and standard errors (ses) are calculated using the available data. A positive outcome means that there is an increase in the incidence of CPSP in the group with preoperative anxiety. ∗In accordance with subgroup analysis, study data from Borges and colleagues^56^ are combined as no effect of the anxiety measurement tool is found. CI, confidence interval; CPSP, chronic post-surgical pain.

After removing the three articles because of a serious risk of bias, 23 articles were included in the meta-analysis. The results are presented in Figure 2 (see Supplementary Table S3 for all SMD data). The overall effect for anxiety was 0.31 with a range of 0.20–0.41 (95% confidence interval [CI]) and *P*<0.01. The estimated standard deviation between studies (Tau^2^) is 0.03. The magnitude of heterogeneity (*I*^2^) is 78% and *P*<0.01, which is considered high.^25^^,^^64^ As shown in the figure, three articles have a negative standard mean difference.^49^^,^^53^^,^^59^ For all other articles included in this meta-analysis, the SMD is positive, although some CIs crossed the zero line. A funnel plot of the included articles (see Supplementary Fig. S2) showed an asymmetric distribution.

**Fig 3:**
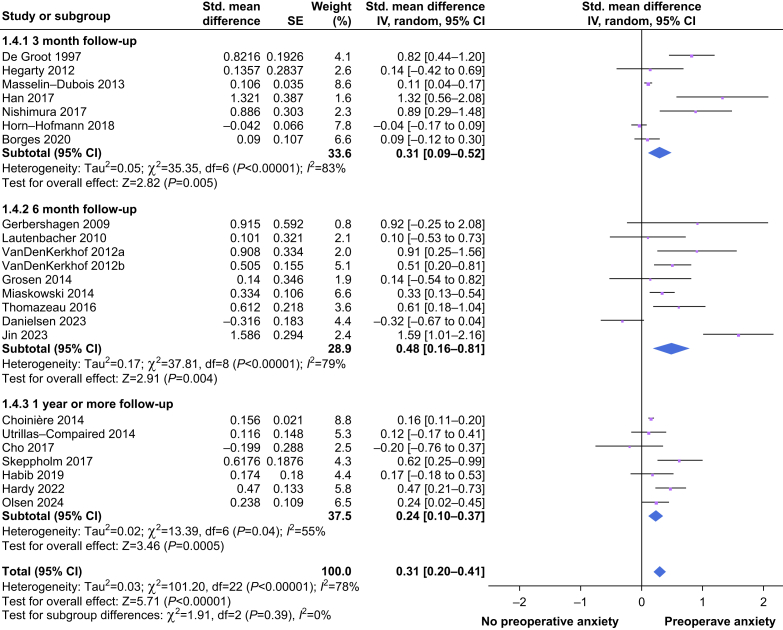
Forest plot of the subgroup analysis on follow-up time after surgery. All standard mean differences (SMDs) and standard errors (ses) are calculated using the available data. A positive outcome means that there is an increase in the incidence of CPSP in the group with preoperative anxiety. CI, confidence interval; CPSP, chronic post-surgical pain.

To investigate a potential effect of follow-up time after surgery, a subgroup analysis was conducted (Fig. 3). For studies with a follow-up duration of 3 months, the overall effect for anxiety was 0.31 (95% CI 0.09–0.52). For a follow-up duration of 6 months, the overall effect was 0.48 (95% CI 0.16–0.81). For a follow-up duration of 1 yr or more, the overall effect was 0.24 (95% CI 0.10–0.37). The magnitudes of heterogeneity were 83%, 79%, and 55%, respectively. Overall, there was a statistically non-significant effect for subgroup differences (*P*=0.39). A subgroup analysis of the anxiety measurement tools (STAI *vs* HADS; Supplementary Fig. S3) yielded a statistically non-significant effect for subgroup differences (*P*=0.65).

### Clinical relevance

For calculating the clinical relevance of the outcome, the SMD generated in the meta-analysis was multiplied by the standard deviation of the relevant scale. For this, the largest and, therefore, most representative trial was selected, which used the NRS pain scale.^60^ The standard deviation of NRS pain in this trial was 1.1, resulting in an overall increase in NRS pain intensity of 0.34 (on a scale of 0–10) in patients with preoperative anxiety compared with patients without preoperative anxiety.

## Discussion

This systematic review and meta-analysis aimed to review studies evaluating the association between preoperative anxiety and CPSP and to evaluate the clinical relevance of this association. The overall meta-analysis found a statistically significant effect of 0.31 (95% CI 0.20–0.41), where the presence of preoperative anxiety is related to increased incidence of CPSP. The outcome of the meta-analysis is estimated to be a small effect as the SMD is <0.5^32^ and a high amount of heterogeneity is present. When translated into a more clinically relevant scale (NRS pain), patients with preoperative anxiety showed a small increase in chronic pain intensity (+0.34 on a scale of 0–10) compared with patients without preoperative anxiety. Following the IMMPACT recommendations on clinical importance,^65^ a change of 10% at the individual level is considered clinically relevant. Therefore, at the individual level, the effect size is small. However, at the group level, smaller differences may also be considered clinically relevant. Another criterion for evaluating the clinical significance of the outcome at the group level is whether a statistically significant difference exists. Given the high amount of heterogeneity in the reviewed literature, the magnitude of clinical relevance on the observed effect is unknown.

The use of non-randomised studies always results in greater heterogeneity than when using randomised trials, because of greater variability in study design and patient characteristics.^66^ Nevertheless, other reasons for heterogeneity are possible. Firstly, there is a high difference in follow-up time between studies, ranging from 3 months to 2 yr. To eliminate the impact of varying follow-up durations, it would be advantageous to present results for each time point measured throughout the study, facilitating an analysis of outcome data at identical follow-up intervals. Furthermore, the incidence and severity of CPSP typically decrease over time after surgery.^67^ However, a subgroup analysis of follow-up time did not show a statistically significant effect and did not reduce heterogeneity. Secondly, there could be an effect of surgery subgroups. Within the included studies, patients underwent a range of different surgeries from various specialties. It could be possible that for certain surgeries, there is an increased risk of preoperative anxiety or CPSP compared with other surgeries. A subgroup analysis for the different surgeries or other patient characteristics was not possible because of the large number of subgroups present. Nevertheless, the study conducted by Masselin-Dubois and colleagues^42^ used two different types of surgery, total knee arthroplasty and breast surgery. They found no evidence that the type of surgery affected the incidence of CPSP. A different result was found in the large cohort study by Jin and colleagues,^60^ which included all patients undergoing elective surgery. Patients who received orthopaedic surgery were associated with a higher risk of CPSP compared with those undergoing other types of surgery. A more extensive study is recommended to define the role of surgery type in the development of CPSP. A third reason for heterogeneity is the possibility of confounders within the different patient groups. In this paper, we excluded patients with existing chronic pain before the procedure, which is known to be an important risk factor for CPSP after surgery. Still, there is a high variety between patients used in the different studies, resulting in the possibility of different risk factors influencing CPSP or preoperative anxiety. Factors influencing CPSP are summarised in a review by Lopes and colleagues.^7^ Another study^68^ found several factors to be associated with preoperative anxiety, such as female sex or education status, suggesting an effect of patient characteristics on both CPSP and anxiety. It is striking that both preoperative anxiety and CPSP share many risk factors, suggesting an interaction between the two. Besides preoperative anxiety, there are many other potential risk factors, such as female sex, age, or educational status. However, because of a lack of available data, conducting a subgroup analysis to examine the potential effects of these risk factors on heterogeneity was not feasible. Among the studies included in this meta-analysis, there was high variability in whether and how known confounders were adjusted for. Future research should control for these confounders to eliminate the influence of patient characteristics on the outcome. Furthermore, in this article, we did not distinguish between different types of preoperative anxiety. It is possible that specific anxieties, such as awareness during surgery or loss of control, may have a more substantial influence than others. In a study where particular causes of anxiety were measured,^13^ the ranking of the specific causes was similar for the high and low anxiety groups, and the difference in anxiety level was similar for each particular cause in the high and low anxiety groups. This suggests that there is no added value to distinguishing between different causes of preoperative anxiety. Taken together, the differences in study design, patient characteristics, and surgical characteristics have contributed to significant heterogeneity, thereby diminishing the reliability of the results of this meta-analysis. For future research, it is recommended to use standardised methods to increase comparability. Furthermore, with a sufficiently large number of participants, it would be possible to perform subgroup analyses based on surgery type and patient characteristics, which could potentially reduce heterogeneity.

One strength of this analysis is the selection of specific tools to measure preoperative anxiety. Theunissen and colleagues^15^ argued in their review that using unreliable anxiety measurements could lead to underreporting the prevalence of anxiety, thereby questioning the reliability of their results. In many cases, the presence of anxiety was estimated by the surgeon or anaesthesiologist and documented in the chart of the patient.^69^^,^^70^ Patients cope with preoperative anxiety in many different ways, and it could, therefore, not be visible from the outside whether they are anxious or not. Both HADS and STAI have been proved valid instruments,^71^, ^72^, ^73^, ^74^ resulting in a more valid and comparable diagnosis of anxiety, thus strengthening the reliability of the meta-analysis. Furthermore, a subgroup analysis by anxiety measurement tool yielded a statistically non-significant and non-relevant change in effect and did not reduce heterogeneity. Another strength of this analysis is the global representation of patients within the studies used. A broad range of countries was included, thereby demonstrating that the management of CPSP is a worldwide issue.

One limitation of this review is the range for the number of participants used in the various studies. In particular, studies with low patient numbers can lead to higher variability. Small studies could show more extreme effects compared with larger studies^75^ and result in a higher sampling error.^76^ However, the inclusion of studies with a high sample size and the use of Cohen’s *d* should lead to a decrease in sample bias,^76^^,^^77^ thus decreasing the effect of a low sample size. Secondly, the scope of this review was limited to preoperative anxiety only. It might also be interesting to investigate the potential influences of intraoperative anxiety by including studies that use local or regional anaesthesia. Next, for this review, we have chosen to use SMDs to be able to include studies using both continuous and correlational data. When converting among effect sizes, the resulting value is always an approximation of the real SMD. Assumptions are made about the underlying data, resulting in a loss of data.^29^ The alternative would be to omit all studies that do not present raw data, which would result in the loss of information and a high bias as a result of sampling error. Furthermore, there may be an effect of publication bias, as studies with positive results are more likely to be published.^78^ Although the funnel plot showed an asymmetric distribution, this might not be attributable to publication bias, but rather a result of baseline risk or sample size.^79^ In this meta-analysis, we used SMDs with continuous data; however, there is currently no reliable or valid method to determine the presence of publication bias for these type of data.^80^^,^^81^ Therefore, it remains uncertain whether the risk of publication bias affects the reliability of the results. Another limitation is the difference in study design between the included papers. In some papers, preoperative anxiety was measured 1 day before surgery.^38^ In other papers, this was up to 1 month before surgery.^60^ Clinicians themselves sometimes administered postoperative pain measurements,^45^ but in other studies, this was administered via post or email.^37^ The lack of consistency between studies could result in high variability or heterogeneity, which decreases the confidence in the meta-analysis. Despite the high heterogeneity found in this review, the meta-analysis remains meaningful because of the comparison of sufficiently homogeneous outcomes.^19^ Furthermore, since the direction of the effect is similar for most articles (21 out of 24), the influence of heterogeneity may be less pronounced.^64^ This gives a strong indication that preoperative anxiety has an association with CPSP, although the magnitude of this influence is less understood.

### Clinical implications

Given the small effect size observed in this meta-analysis, it is not possible to draw firm conclusions about the clinical implications at the individual level. Given that the overall effect showed a statistically significant difference, which is one of the considerations for the clinical importance of group differences, in line with IMMPACT recommendations, the implementation of anxiety reduction treatments could potentially lead to a decrease in CPSP. A previous review^82^ on the effect of psychological interventions for anxiety and stress found a postoperative reduction in opioid use and pain scores in some studies; however, more research is needed to strengthen these results. Furthermore, preoperative patient education,^83^^,^^84^ virtual reality,^85^ and cognitive behavioural therapy^86^^,^^87^ are studied as potential therapies for reducing preoperative anxiety and postoperative pain. However, the evidence of these preventive strategies remains inconclusive because of small study groups, differences in methodology, and short follow-up time. More research is needed, particularly regarding the impact of anxiety reduction on CPSP.

In conclusion, this meta-analysis shows that there is moderate-quality evidence for a small effect of preoperative anxiety on CPSP after general anaesthesia. The variability in the strength of this effect appears to be dependent on high heterogeneity in study characteristics, such as the type of surgery, study design variation, or other confounding factors. As the treatment of CPSP is complex, preventive approaches may be more successful. When factors can be identified that are most strongly associated with the development of CPSP, creating specific interventions to reduce the effect of these factors could potentially reduce the incidence of CPSP. We advise focusing research on patient groups with risk factors for both preoperative anxiety and CPSP. This will enable the creation of targeted interventions to reduce CPSP, tailored to the unique needs of each individual. By focusing on preventing anxiety, the incidence of CPSP could be reduced. This would increase patients’ welfare while reducing the healthcare burden.

## Authors’ contributions

Conceptualisation: MBB, AW

Methodology: MBB

Literature search: MBB, RH, RS

Validation of data: all authors

Formal analysis, writing of the original manuscript: MBB

Review of manuscript: RH

Validation of analysis: RS

Review and editing of manuscript, supervision: RS, AW

## Declarations of interest

RS is Editor-in-Chief of the *Journal of Back and Musculoskeletal Rehabilitation*. All other authors declare that they have no conflicts of interest.
