# Supplementary material for: The association between preoperative anxiety and chronic post-surgical pain after general anaesthesia, a systematic review and meta-analysis
Source: BJA Open. 2025 Oct 6;16:100487. doi: 10.1016/j.bjao.2025.100487 (PMC12538085; doi:10.1016/j.bjao.2025.100487)

Supplemental content

**Manuscript title:**

*The association between preoperative anxiety and chronic post-surgical pain after general anaesthesia, a systematic review and meta-analysis.*

**Content:**

Supplemental table S1: search strategy

Supplemental table S2: confounder adjustments

Supplemental table S3: standardized mean differences

Supplemental figure S1: Risk of Bias scoring for each paper

Supplemental figure S2: Funnel plot

Supplemental figure S3: Subgroup analysis of anxiety measurement tool

Supplemental Table S1: search strategy

| **Database** | **Search Strategy** | **Results** |
| --- | --- | --- |
| PubMed | ("Preoperative Period"[Mesh] OR Preoperat*[tiab] OR "pre-operat*"[tiab] OR "pre-surg*"[tiab] OR presurg*[tiab]) AND (Anxiety) AND pain AND ("Postoperative Period"[Mesh] OR postoperat*[tiab] OR post-operat* OR postsurg*[tiab] OR post-surg*[tiab]) | 1272 records |
| EMBASE | preoperative:ab,ti AND anxiety:ab,ti AND postoperative:ab,ti AND pain:ab,ti | 1877 records  (1241 duplicate with PubMed) |
| PsychINFO | preoperative anxiety or pre-operative anxiety or pre-surgical anxiety  AND  postoperative or post operative or post-surgery or post-surgical  AND  chronic pain or persistent pain or long term pain | 22 records  (4 duplicate with PubMed) |
| Cochrane Library | preoperative in Title Abstract Keyword AND anxiety in Title Abstract Keyword AND "postoperation" in Title Abstract Keyword AND "chronic pain" in Title Abstract Keyword - (Word variations have been searched) | 0 records |

Supplemental table S2: Confounder adjustments for the included studies in the meta-analysis

| Table S3 Effect of different confounders | | | | | |
| --- | --- | --- | --- | --- | --- |
| First author | Year | Effect of different confounders (p-value) | | | |
|  |  | *Age* | *Sex* | *Education* | *Overweight* |
| De Groot | 1997 | n.s. | n.s. |  | n.s. |
| Gerbershagen | 2009 |  |  |  |  |
| Lautenbacher | 2010 |  | male only |  |  |
| Hegarty | 2012 | 0.41 | 0.56 |  |  |
| VanDenKerkhof | 2012a |  | female only | n.s. | n.s. |
| VanDenKerkhof | 2012b | n.s. |  |  | n.s. |
| Masselin-Dubois | 2013 | 0.03 | 0.34 |  |  |
| Choinière | 2014 | n.s. | 0.05 | n.s. | n.s. |
| Grosen K | 2014 |  |  |  |  |
| Miaskowski C | 2014 | 0.01 | female only | n.s. | 0.01 |
| Utrillas-Compaired | 2014 | 0.04 | 0.07 |  | 0.55 |
| Thomazeau | 2016 | 0.09 | 0.10 | 0.01 | 0.27 |
| Cho | 2017 | 0.40 | 0.45 |  |  |
| Han | 2017 | 0.38 | female only | 0.74 | 0.59 |
| Nishimura | 2017 | 0.27 | female only |  | 0.71 |
| Skeppholm | 2017 |  | 0.36 |  |  |
| Horn-Hofmann C | 2018 |  |  |  |  |
| Habib | 2019 | 0.01 | female only | 0.38 | 0.77 |
| Borges | 2020 | 0.13 | female only | 0.86 |  |
| Hardy | 2022 | 0.57 | 0.16 |  | 0.14 |
| Danielsen | 2023 | 0.09 | n.s. |  | n.s. |
| Jin | 2023 | 0.38 | 0.857 |  | 0.38 |
| Olsen | 2024 | 0.85 | 0.043 |  |  |
| P-value is effect of confounder on outcome (pain after > 3 months); n.s. = not significant (no p-value given) | | | | | |

Supplemental table S3: standardized mean differences

| **Table S2** Dataset for calculated Standardized Mean Differences (SMDs) | | | | | | | |
| --- | --- | --- | --- | --- | --- | --- | --- |
| **First author** | **Year** |  | **Cohen’s *d***  **(calculated)** | **95% CI Upper** | **95% CI lower** | | **SE**  **(calculated)** |
| De Groot | 1997 |  | 0.82 | 1.20 | 0.44 | | 0.19 |
| Gerbershagen | 2009 |  | 0.92 | 2.12 | -0.29 | | 0.59 |
| Lautenbacher | 2010 |  | 0.10 | 0.50 | -0.77 | | 0.32 |
| Hegarty | 2012 |  | 0.37 | 0.91 | -0.18 | | 0.27 |
| VanDenKerkhof | 2012a |  | 0.91 | 1.57 | 0.24 | | 0.33 |
| VanDenKerkhof | 2012b |  | 0.51^$^ | 0.81 | 0.20 | | 0.16 |
| Masselin-Dubois | 2013 |  | 0.11 | 95% CI from OR | | | 0.04 |
| Choinière | 2014 |  | 0.16 | 95% CI from OR | | | 0.02 |
| Grosen | 2014 |  | 0.14 | 0.85 | | -0.57 | 0.35 |
| Miaskowski | 2014 |  | 0.33 | 0.54 | | 0.13 | 0.11 |
| Utrillas-Compaired | 2014 |  | 0.12 | 0.41 | -0.18 | | 0.15 |
| Thomazeau | 2016 |  | 0.61 | 1.04 | 0.18 | | 0.22 |
| Cho | 2017 |  | -0.20 | 0.38 | -0.78 | | 0.29 |
| Han | 2017 |  | 1.32 | 95% CI from OR | | | 0.39 |
| Nishimura | 2017 |  | 0.89 | 95% CI from OR | | | 0.30 |
| Skeppholm | 2017 |  | 0.62 | 0.99 | 0.25 | | 0.19 |
| Horn-Hofmann | 2018 |  | -0.04 | 95% CI from OR | | | 0.07 |
| Habib | 2019 |  | 0.17 | 0.53 | -0.18 | | 0.18 |
| Borges | 2020 |  | 0.09^$^ | 0.30 | -0.12 | | 0.11 |
| Hardy | 2022 |  | 0.47 | 0.73 | 0.21 | | 0.13 |
| Danielsen | 2023 |  | -0.32 | 0.04 | -0.68 | | 0.18 |
| Jin | 2023 |  | 1.59 | 95% CI from OR | | | 0.29 |
| Olsen | 2024 |  | 0.24 | 95% CI from OR | | | 0.11 |
| CI = Confidence Interval; SE = Standard Error; OR = Odds Ratio; n.s. = not significant; $ = 2x2 table derived from text | | | | | | | |

Supplemental figure S1 Risk of Bias scoring for each paper


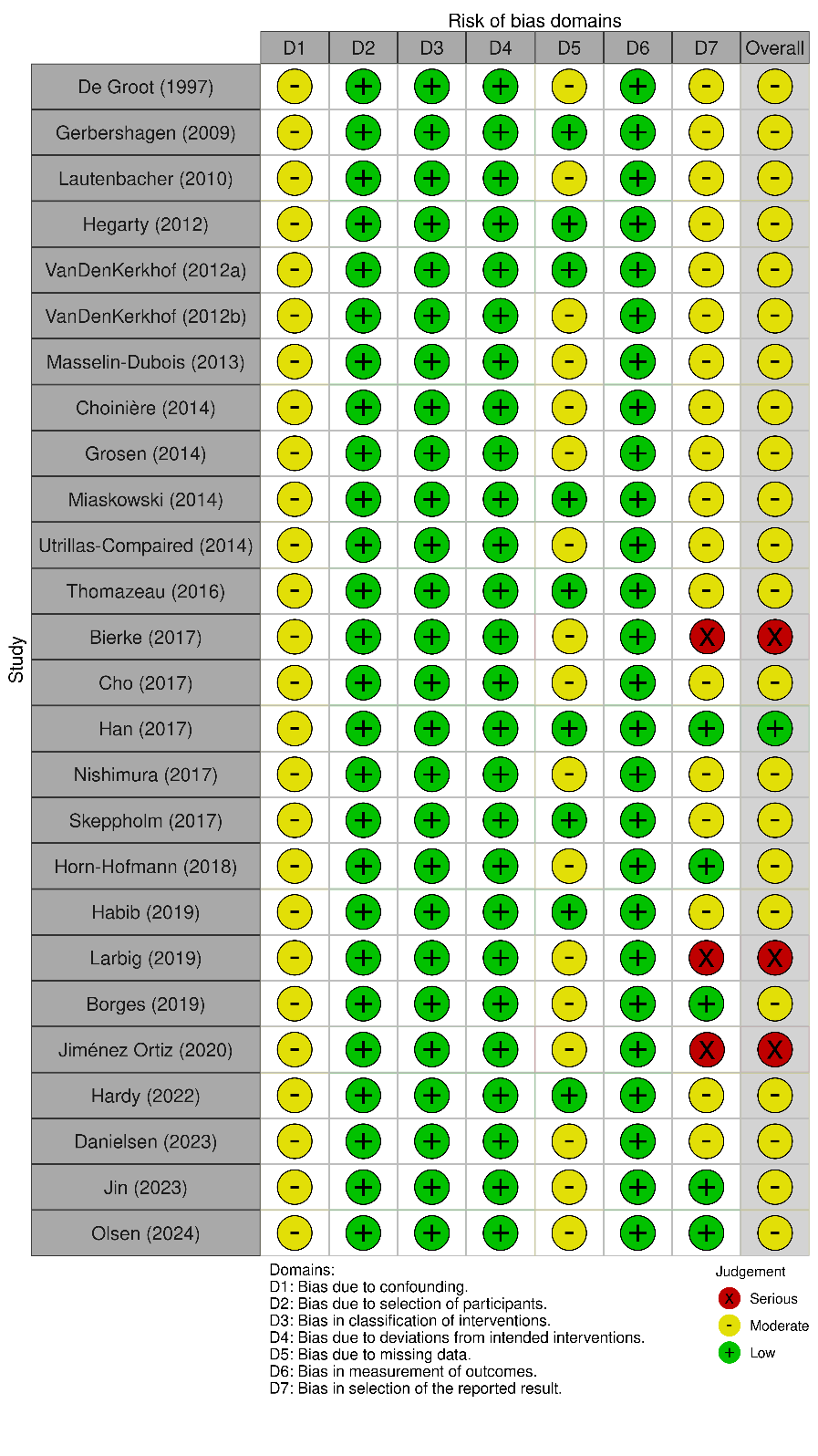


Supplemental figure S2 Funnel plot


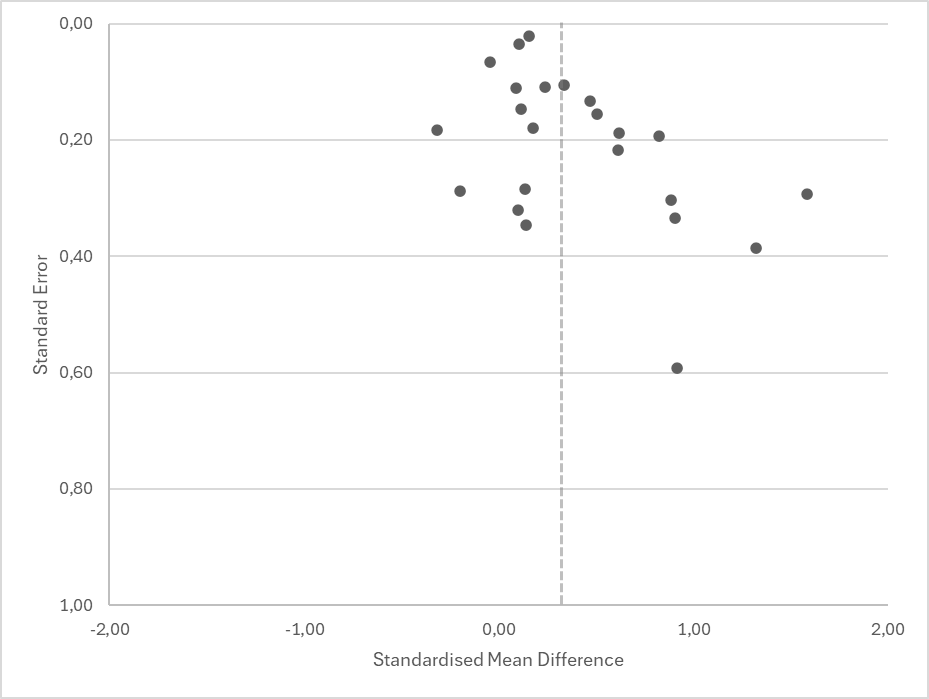
Supplemental figure S3 Subgroup analysis of anxiety measurement tool


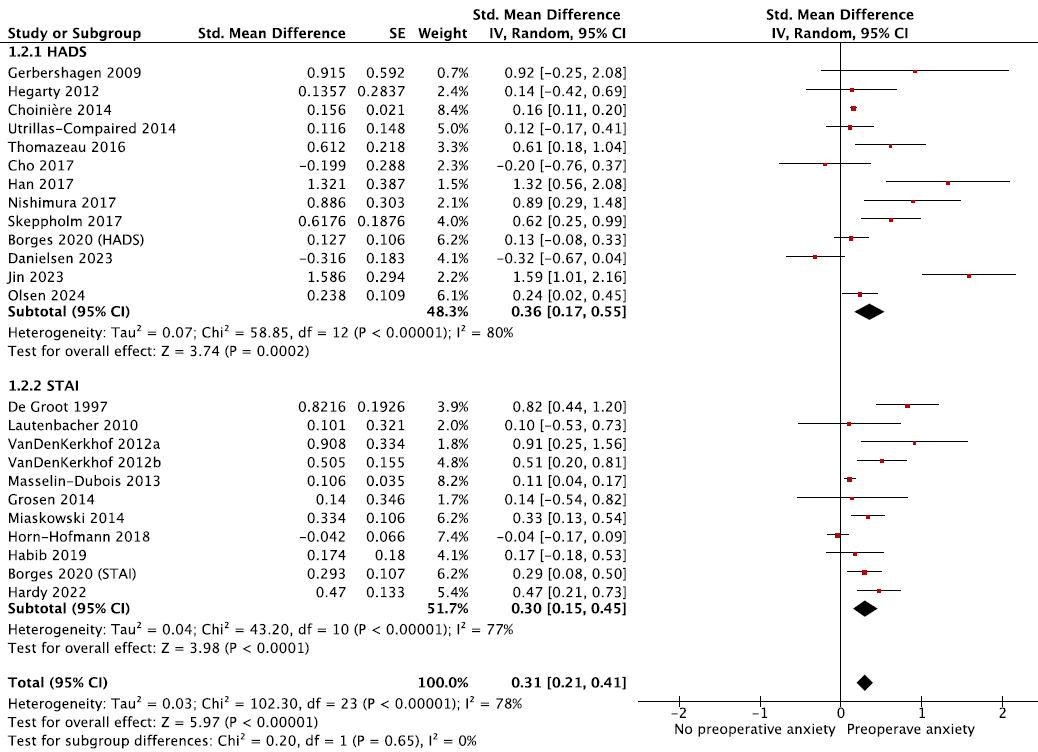

Supplement: Multimedia Component 1 [file mmc1.docx]
